# FOSTERING SOCIAL CONNECTIVITY AND RESILIENCE IN SEXUAL AND GENDER MINORITY OLDER ADULTS

**DOI:** 10.1093/geroni/igac059.1419

**Published:** 2022-12-20

**Authors:** Hyun-Jun Kim

## Abstract

While social connectedness and engagement are key aspects of well-being over the life course, sexual and gender minority (SGM) older adults are found to experience elevated risks of social isolation and limited social connectivity, which have been linked to loneliness. Social isolation and loneliness have been significant health concerns, increasing risk of premature death, cognitive decline, and poor health and well-being, yet SGM older adults are resilient populations. To address the strengths and challenges found in evidence-based intervention development, it is critical to understand how they have managed to build and maintain their social resources and reduce social isolation and loneliness. To this end, this symposium will analyze and incorporate the results using data from both Aging with Pride: National Health, Aging and Sexuality/Gender Study (NHAS), the first longitudinal study of SGM older adults in the United States and Innovations in Empowerment and Action (IDEA) Café, the first clinical trial pilot study addressing social isolation among SGM older adults living with dementia. Dr. Kim and colleagues with present a factor analysis suggesting a multi-dimensional construct of social resources unique to SGM older adults. Dr. Fredriksen Goldsen and colleagues will identify sexual and gender diverse older adults at heightened risk of loneliness and examine risk and protective factors. Dr. Emlet and colleagues will present findings from a pilot study, IDEA Café, designed to enhance social connectedness, physical functioning, and quality of life among socially isolated SGM older adults with cognitive impairment.

